# PS-40 - EMERGING AND RE-EMERGING STIS IN GAY, BISEXUAL, AND OTHER MEN WHO HAVE SEX WITH MEN (GBMSM) IN ENGLAND

**DOI:** 10.1093/pubmed/fdag046.076

**Published:** 2026-07-09

**Authors:** Miss Megan Walsh, Holly Fountain, Katy Sinka

**Affiliations:** UK Health Security Agency; UK Health Security Agency; UK Health Security Agency

## Abstract

**Background:**

Gay, bisexual, and other men who have sex with men (GBMSM) have been disproportionately affected by emerging/re-emerging STIs: lymphogranuloma venereum (LGV), syphilis, and shigella, each of which increased over time to become endemic. Real-time enhanced STI surveillance supports detection, monitoring, and control. Understanding past emergence may help guide responses to new challenges such as mpox.

**Objectives:**

Describe trends and epidemiology of emerging STIs among GBMSM in England to inform understanding of current and future infections and prevention options.

**Methods:**

Infectious syphilis and LGV diagnoses among GBMSM ≥15 years were extracted from GUMCAD (2010–2024). Shigella (2010–2024) and mpox (from 2022) data were obtained from SGSS. Adult males without reported travel were used as a proxy for sexually transmitted shigella; mpox data included GBMSM and adult males without recorded sexual orientation. Descriptive analyses were conducted separately as datasets could not be linked. Published surveillance data summarised trends before 2010.

**Results:**

Syphilis re-emerged and LGV emerged in 2003, followed by sexually transmitted shigella in 2008. All increased markedly between 2010–2019: LGV: 217–1,053, +385%; syphilis: 1,610–5,877, +265%; shigella: 714–1,309, +83%, particularly following wider HIV-PrEP use from 2017. Post-COVID increases were observed for all infections, steepest for shigella (2021–2024: 642–2,317, +261%). Geographic patterns were consistent, with reports concentrating in London (2024: LGV 66%, syphilis 49%, shigella 54%, mpox 80%), and secondary contributions from the North West and South East. Age distributions were similar, with most diagnoses among 25–34-year-olds, although shigella disproportionately affected older GBMSM.

**Conclusions:**

The overlapping epidemiology of these infections, and shared determinants of transmission and escalation highlight the need for sustained surveillance and syndemic approaches to predict and respond to current and future outbreaks, including mpox. Understanding emergence/re-emergence provides a foundation for anticipating how interventions such as doxyPEP may shift future epidemiology.

